# Significance of hyperuricemia in immunoglobulin A nephropathy

**DOI:** 10.12861/jrip.2013.33

**Published:** 2013-09-01

**Authors:** Hamid Nasri, Mohammad Reza Ardalan

**Affiliations:** ^1^Department of Nephrology, Division of Nephropathology, Isfahan University of Medical Sciences, Isfahan, Iran; ^2^Chronic Kidney Disease Research Center, Tabriz University of Medical Sciences, Tabriz, Iran

**Keywords:** IgA nephropathy, Hyperuricemia, Allopurinol

**Implication for health policy/practice/research/medical education:**

It is well known that hyperuricemia is an independent risk factor for renal progression in IgA nephropathy, however it is unclear the effect of allopurinol therapy, on the clinical outcome in hyperuricemic IgAN. It is possible that appropriate treatment by allopurinol be a reasonable modality in these patients. However, to better understand the kidney protective efficacy of allopurinol in IgAN patients, more clinical studies are suggested.

Immunoglobulin A nephropathy is the most common glomerulonephritis globally (1). IgA nephropathy (IgAN) is an autoimmune disease with a known antigen, galactose-deficient IgA_1_, which can elicit an autoantibody response and formation of immune complexes that are deposited in the mesangial area (2). In histopathology, IgAN is defined by expansion of the mesangial matrix with mesangial cell proliferation and/or mononuclear cell infiltration. Glomeruli contain mainly mesangial deposits of IgA in association with C3 (1-3). Clinically, patients with IgA nephropathy show microscopic and macroscopic hematuria and/or proteinuria. Although the clinical course is generally gradual in patients with IgA nephropathy, progression to renal hypertension, renal anemia, and end-stage kidney disease is not as rare as originally thought. Indeed, in various patient populations, its prognosis can be totally different, ranging from asymptomatic, slow progression to chronic kidney disease and end-stage renal failure in as much as 40% of patients in few months to years. Various risks had been described, which can predict the long-term outcome. Since pathogenesis and radical treatment for IgA nephropathy are still not established, it is necessary to study aggravating factors using various biochemical findings. Thus, it appears that early screening and subsequent intervention are important for a good prognosis in IgAN patients (2-4). Various markers for poor prognosis in IgAN are as follows: (1) heavy proteinuria (2) renal dysfunction at the time of renal biopsy, (3) low serum albumin, (4) male sex, (5) hypertension, (6) age below 30 years. Indeed various studied have addressed the clinical and morphological risk factors related to the risk of IgAN progression. Recently much attention has been directed toward the aggravating effect of hyperuricemia on IgAN (3-5). Uric acid is the final oxidation product of purine catabolism and around 70% of uric acid is eliminated by the kidneys (2-6). Many investigation support the hypothesis that elevated uric acid levels might have a harmful effect, leading to dysfunction of endothelial cells, inflammation vasculopathy. Recent studied showed that the serum uric acid level is closely associated with hypertension in hyperuricemic patients and also with the beginning of hypertension (3-6). In fact some clinical findings have shown that, lowering uric acid with allopurinol ameliorated endothelial dysfunction in both hyperuricemic subjects and even hypertensive type 2 diabetic patients with normal uric acid levels (3-6). The mechanism by which elevated uric acid causes endothelial dysfunction, is inhibiting nitric oxide synthetase, activating the renin–angiotensin system and causing proinflammation and resultant endothelial dysfunction (6). Hyperuricemia is also prevalent in patients with chronic kidney disease. In fact, various investigations have shown that hyperuricemia may have a pathogenic role in the development and progression of chronic kidney disease, rather than simply reflecting decreased renal uric acid excretion (1-6). In the study conducted by Sulikowska *et al*. on 46 non-nephrotic IgAN patients and 15 control subjects with a glomerular filtration rate of 86.7±17.4 and 118.1±17.2 ml/min, respectively, found a greater renal vasoconstriction in IgAN patients (7). Likewise, in the study conducted by Cheng *et al*. prevalence of glomerular sclerosis, vasculopathy and tubulointerstitial fibrosis found to be greater in patients with high serum uric acid in comparison to the patients with normal serum uric acid level. They suggested that serum uric acid level in IgAN affects the pathophysiology and prognosis of the IgAN (8). To study the correlation of serum uric acid and the clinical and morphological features of IgAN, Cui *et al*. on 148 patients with IgAN found that, the level of serum uric acid had correlated with 24-hour proteinuria, level of blood pressure and kidney function in IgAN. They also found, tubulointerstitial damage and pathologic lesions of renal artery were severe in hyperuricemic patients (9). Moreover, Kovács *et al*. in a study on two hundred and twenty three patients with IgAN (107 with and 116 without metabolic syndrome) found that, IgAN patients with metabolic syndrome was significantly correlated with the primary renal end point. They found, hyperuricemia is an independent risk factor of progression of IgAN (10). While it is well known that hyperuricemia is an independent risk factor for renal progression in IgAN, however it is unclear, the effect of allopurinol therapy on the clinical outcome in hyperuricemic IgAN. In a recent study by shi *et al*. 40 hyperuricemic IgAN patients who were randomized to receive allopurinol (100–300 mg/day) or usual therapy for 6 months. They found, hyperuricemia predicts the progression of IgAN independently of baseline estimated glomerular filtration rate and allopurinol may improve the control of blood pressure (11). Thus, it is possible that appropriate treatment by allopurinol be a reasonable modality in these patients (11,12). However, to better understand the kidney protective efficacy of allopurinol in IgAN patients, more clinical studies are suggested.

## Authors’ contributions

MRA and HN wrote the manuscript equally.

## Conflict of interests

The authors declared no competing interests.

## Ethical considerations

Ethical issues (including plagiarism, misconduct, data fabrication, falsification, double publication or submission, redundancy) have been completely observed by the authors.

## Funding/Support

None.
